# Whole-Genome Sequencing to Predict *Mycobacterium tuberculosis* Drug Resistance: A Retrospective Observational Study in Eastern China

**DOI:** 10.3390/antibiotics12081257

**Published:** 2023-07-31

**Authors:** Mingwu Zhang, Yewei Lu, Yelei Zhu, Kunyang Wu, Songhua Chen, Lin Zhou, Fei Wang, Ying Peng, Xiangchen Li, Junhang Pan, Bin Chen, Zhengwei Liu, Xiaomeng Wang

**Affiliations:** 1 Zhejiang Provincial Center for Disease Control and Prevention, Hangzhou 310051, China; mwzhang@cdc.zj.cn (M.Z.); ylzhu@cdc.zj.cn (Y.Z.); kywu@cdc.zj.cn (K.W.); shchen@cdc.zj.cn (S.C.); lzhou@cdc.zj.cn (L.Z.); feiwang@cdc.zj.cn (F.W.); ypeng@cdc.zj.cn (Y.P.); jhpan@cdc.zj.cn (J.P.); bchen@cdc.zj.cn (B.C.); 2 Key Laboratory of Precision Medicine in Diagnosis and Monitoring Research of Zhejiang Province, Hangzhou 310020, China; lyw@cwmda.com (Y.L.); lxc@cwmda.com (X.L.)

**Keywords:** pulmonary tuberculosis, *MTB*, WGS, DST, sensitivity, specificity, positive predictive value, negative predictive value

## Abstract

Pulmonary tuberculosis (TB) is an infectious disease caused by *Mycobacterium tuberculosis* (*MTB*). Whole-genome sequencing (WGS) holds great promise as an advanced technology for accurately predicting anti-TB drug resistance. The development of a reliable method for detecting drug resistance is crucial in order to standardize anti-TB treatments, enhance patient prognosis, and effectively reduce the risk of transmission. In this study, our primary objective was to explore and determine the potential of WGS for assessing drug resistance based on genetic variants recommended by the World Health Organization (WHO). A total of 1105 *MTB* strains were selected from samples collected from 2014–2018 in Zhejiang Province, China. Phenotypic drug sensitivity tests (DST) of the anti-TB drugs were conducted for isoniazid (INH), rifampicin (RFP), streptomycin, ethambutol, fluoroquinolones (levofloxacin and moxifloxacin), amikacin, kanamycin, and capreomycin, and the drug-resistance rates were calculated. The clean WGS data of the 1105 strains were acquired and analyzed. The predictive performance of WGS was evaluated by the comparison between genotypic and phenotypic DST results. For all anti-TB drugs, WGS achieved good specificity values (>90%). The sensitivity values for INH and RFP were 91.78% and 82.26%, respectively; however, they were ≤60% for other drugs. The positive predictive values for anti-TB drugs were >80%, except for ethambutol and moxifloxacin, and the negative predictive values were >90% for all drugs. In light of the findings from our study, we draw the conclusion that WGS is a valuable tool for identifying genome-wide variants. Leveraging the genetic variants recommended by the WHO, WGS proves to be effective in detecting resistance to RFP and INH, enabling the identification of multi-drug resistant TB patients. However, it is evident that the genetic variants recommended for predicting resistance to other anti-TB drugs require further optimization and improvement.

## 1. Introduction

Tuberculosis (TB) is an infectious disease caused by *Mycobacterium tuberculosis* (MTB). According to the “Global Tuberculosis Report” published by the World Health Organization (WHO) in 2022, 10.6 million new cases of TB worldwide were estimated in 2021, and 1.4 million people died from the disease. That year, 3.6% of new patients and 18% of retreated patients developed RFP-resistant or multidrug-resistant tuberculosis (MDR-TB), and 450,000 patients developed drug-resistant TB (DR-TB) [1]. DR-TB, especially MDR-TB, poses many problems during diagnosis and treatment and is one of the main challenges affecting global TB prevention and control [2,3,4].

Regarding the diagnosis of DR-TB, traditional phenotypic drug sensitivity testing (DST) is an effective tool; however, it requires a long testing time (>2 weeks), and the stability of some drugs needs to be improved. The lack of rapid diagnostic and detection tools for drug resistance hinders the formulation of standard treatment regimens based on DST results for patients with DR-TB, especially MDR-TB. Non-standard anti-TB treatment also leads to related issues such as the spread and variation of drug-resistance patterns [5]. This is an important factor that contributes to the poor prognosis of patients with DR-TB. Molecular diagnostic technologies can overcome the shortcomings of conventional phenotypic DST methods [6].

Molecular diagnostic tools possess advantages in *MTB* detection, such as fast detection speed, high sensitivity, and reasonable specificity [7]. Based on the molecular mechanism that attributed the majority of drug resistance in clinical *MTB* strains to chromosomal mutations or epigenetic modifications, a series of molecular tools, such as GeneXpert, have been developed that are helpful for the detection of anti-TB drug resistance. However, most methods can only detect resistance to individual anti-TB drugs. Newly developed molecular tools may effectively improve the clinical diagnosis of TB, partly [8], improving the clinical treatment effectiveness of some TB patients [9], and increasing the treatment success rate of DR-TB [10]. With the advancement of technology, molecular diagnostics have gradually expanded to predict common anti-TB drug resistance [11]. However, molecular methods are limited in their prediction of sensitivity to other common anti-TB drugs, especially second-line drugs for MDR-TB patients [12].

Whole-genome sequencing (WGS) technology has shown promising applications in TB detection and the prediction of anti-TB drug resistance. For example, Wu et al. designed a customized Ion AmpliSeq TB analysis platform that can obtain analysis results consistent with the phenotypic DST and effectively shorten the diagnosis time [13]. Furthermore, WGS technology is a useful tool for monitoring genetic variations and mutations in TB caused by abnormal anti-TB treatment and clinical drug use [14] and for the early detection and prevention of MDR-TB and extensively drug-resistant tuberculosis (XDR-TB) [15].

WHO recommends a series of mutation sites for each of the common anti-TB drug-resistance predictions using WGS (Table S1). However, the predictive value of these mutated loci needs to be validated with further studies involving different populations and regions. In the present study, WGS was performed among patients with common pulmonary tuberculosis (PTB), based on a population-based study conducted in Zhejiang Province, China, from 2014 to 2018, and aimed to provide a theoretical basis and technical support for DR-TB diagnosis and treatment.

## 2. Results

### 2.1. Phenotypic DST Results

A total of 1105 *MTB* strains were included in this study. As shown in Table 1, the highest resistance rate is observed for streptomycin (SM) (14.39%, 159/1105). This was followed by isoniazid (INH) (11.22%, 124/1105), rifampicin (RFP) (6.60%, 73/1105), ethambutol (EMB) (5.70%, 63/1105), levofloxacin (LFX) (5.43%, 60/1105), kanamycin (KM) (4.80%, 53/1105), and capreomycin (CM) and moxifloxacin (MFX) with the same resistance rate (2.53%, 28/1105). The lowest resistance rate was observed for amikacin (AK) (0.05%, (5/1105).

### 2.2. Predictive Value of WGS Genotypic DST

The results of WGS genotypic DST for different anti-TB drugs are shown in Table 2. WGS achieved good specificity values in predicting resistance to all anti-TB drugs, which were above the 95% level. The sensitivity values for RFP and INH were 91.78% and 82.26%, respectively; however, the sensitivity values for other anti-TB drugs were only 60% or less.

### 2.3. Distributions of Drug Resistance-Associated Mutations

**Figure 1:**
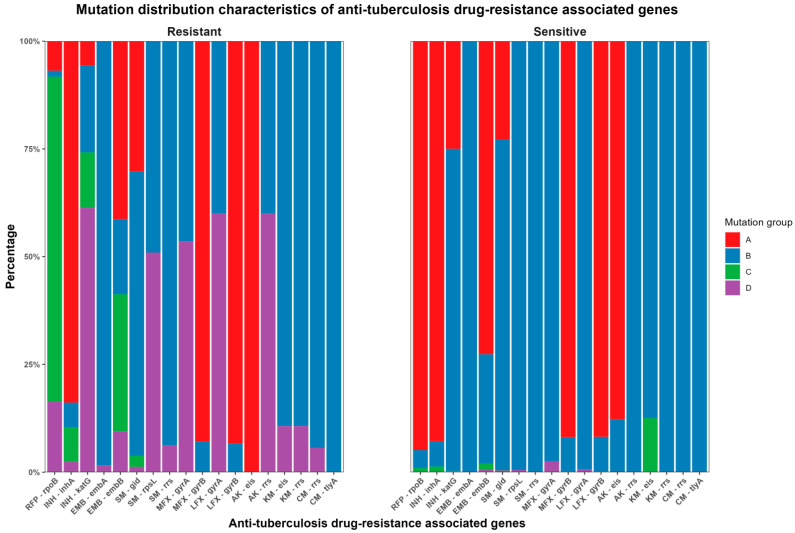
Distribution characteristics of genetic mutation sites related to anti-TB drug resistance.

Based on the WHO recommendations and anti-TB drug-resistance-associated gene mutations detected by WGS (Table S2), the MTB strains were classified into four categories: A: no mutations; B: only mutations other than WHO-recommended ones; C: only mutations recommended by WHO; and D: mutations included in both WHO-recommended ones and others. The distribution of these categories for different drugs is shown in Table 3 and Figure 1.

In phenotypically sensitive strains, WGS revealed that no mutations were detected in 94.9% (979/1032) of rpoB, 92.8% (910/981) of inhA, 25.0% (245/981) of katG, 72.6% (757/1042) of embB, 22.8% (216/946) of gid, 91.8% (989/1077) of MFX-resistance-related gyrB, 91.8% (959/1055) of LFX-resistance-related gyrB, and 87.7% (965/1100) of eis. The detected mutations primarily occurred in Group B, whereas they were relatively less frequent in Groups C and D (containing WHO-recommended mutations). The highest proportion was observed for eis mutations associated with KM resistance at 12.5% (135/1077). This was followed by gyrA mutations related to MFX resistance at 2.6% (28/1077). Moreover, the frequencies of other mutations were less than 1.5%.

In phenotypically resistant strains, 6.9% (5/73) of rpoB, 83.9% (104/124) of inhA, 5.6% (7/124) of katG, 41.4% (26/63) of embB, 30.2% (48/159) of gid, 92.9% (26/28) of MFX-resistance-related gyrB, 93.3% (56/60) of LFX-resistance-related gyrB, and 100% (5/5) of AK-resistance-related eis showed no mutations. Among the drug-resistant strains with detected mutations, 1.3% (1/73) of rpoB, 5.6% (7/124) of inhA, 20.2% (25/124) of katG, 98.4% (62/63) of embA, 17.5% (11/63) of embB, 66.0% (105/159) of gid, 49.1% (78/159) of rpsL, 93.7% (149/159) of SM-resistance-related rrs, 46.4% (13/28) and 7.1% (2/28) of MFX-resistance-related gyrA and gyrB, respectively, 40% (2/5) of AK-resistance-related rrs, 89.3% (25/28) of KM-resistance-related eis and rrs, and 94.3% (50/53) and 100% (53/53) of CM-resistance-related rrs and tlyA, respectively, were unrelated to the WHO-recommended mutations. Additionally, other mutations were partially or entirely within the recommended WHO mutations.

As shown in Table S1, except for AK, KM, and CM, mutations associated with other anti-TB drug resistance coexisted in the phenotypically resistant and sensitive groups. Meanwhile, cases exist where mutations are not detected in drug-resistant strains but are detected in sensitive groups. These cases, in terms of each anti-TB drug, are described as follows:

- RFP: Of the 73 RFP-resistant strains, six did not have any mutations. Forty-two strains were detected with the rpoB_p. Ser450Leu site, which had the highest mutation frequency.
- INH: Among the 124 INH-resistant strains, no mutations were detected in 22 strains. The katG_p. Ser315Thr site was detected in 85 strains, which had the highest mutation frequency.
- EMB: Of the 63 EMB-resistant strains, 37 did not contain any mutations. Eleven strains were detected with the embB_p. Met306Val site, which had the highest mutation frequencies.
- LFX: Among the 60 LFX-resistant strains, mutations were not detected in 24. In 15 strains, gyrA_p. mutations were detected at the Asp94Gly site, showing the highest frequency, followed by gyrA_p. Ala90Val (11 strains) and gyrA_p. Asp94Asn (7 strains).
- MFX: For MFX-resistant strains, gyrA_p. Ala90Val and Asp94Gly were the two sites with the highest mutation rates, with three and eight strains in the drug-resistant group, respectively, and 11 and 10 strains in the sensitive group, respectively.
- SM: Out of 159 SM-resistant strains, no mutations was detected in 63 strains; Out of the detected mutant strains, 72 strains showed mutations in the rpsL_p. Lys43Arg site.
- AK: Of the five resistant strains, no mutation was detected in two strains, and the rrs_n.1401A>G mutation was detected in three strains.
- KM: No mutations were detected in 22 of the 28 KM-resistant strains. Three, two, and one strains were detected with mutations at rrs_n.1401A>G, eis_c.-37G>T, and eis_c.-10G>A, respectively.
- CM: Among the 53 resistant strains, no mutations were detected in 50 strains and three were detected with mutated rrs_n.1401A>G.

## 3. Discussion

DR-TB, particularly MDR-TB, is a major concern in TB prevention and control. Phenotypic anti-TB DSTs play a key role in the early detection of DR-TB [16] and provide a basis for patients to receive standardized anti-TB treatment, thereby improving its efficacy [17]. Standardized anti-TB treatment is also crucial to reducing the spread of DR-TB. Recently, the application of WGS technology for predicting drug resistance has received widespread attention [18,19]. It covers the most common anti-TB drugs and provides timely detection results. Therefore, WGS has the potential for the rapid detection of drug-resistant MTB strains, leading to more anti-TB drugs and supporting TB treatment [20]. Based on the progress in WGS, the WHO has selected a series of genetic mutations to predict drug resistance.

Based on the mutation sites recommended by the WHO, this study was conducted in a Chinese population with TB. We found that WGS can effectively identify non-resistant strains for most anti-TB drugs as well as RFP- and INH-resistant *MTB* strains among typical PTB patients. However, the predictive effect for EMB, SM, AK, LFX, MFX, KM, and CM resistance could be improved.

In the present study, the prediction of RFP resistance based on WHO-recommended mutation sites achieved a sensitivity and specificity of over 90%, and this prediction was closely related to the mutation of key sites in the *rpoB* gene for RFP resistance [21]. Current sequencing technologies can detect common genetic mutations; however, different genetic variations lead to different characteristics of RFP resistance [22]. The study also found that 75.3% of *rpoB* mutations in RFP-resistant strains only occurred at WHO-recommended loci, whereas 16.4% of mutations occurred simultaneously at sites outside the recommended ones. Thus, approximately 92% of the resistant strains have mutations at the WHO-recommended loci, whereas this proportion in the sensitive strains is only 0.9%. In INH-resistant strains, the proportion of WHO-recommended mutations in *inhA* and *katG* genes, which are closely related to INH resistance, was approximately 80%, whereas the mutation frequency of the corresponding sites in sensitive strains was approximately 1.3%.

Finci et al. found that WGS is highly accurate in predicting resistance to INH and can achieve good sensitivity and specificity for LFX, KM, AK, and Cs, but the results are not ideal for RFP, pyrazinamide (PZA), and EMB [23]. A study in China showed that WGS had good predictive results for common anti-TB drugs, with overall sensitivity and specificity values, respectively, of 97.1% and 90.4% for RFP, 91.0% and 95.2% for INH, 100.0% and 87.3% for EMB, 86.5% and 95.2% for OFX, 100.0% and 67.6% for AK, 100.0% and 67.2% for KM, and 62.5% and 88% for PZA [24]. Another study conducted in Shanghai, China, showed that the concordance between WGS genotypic and phenotypic DST results for AK/KM, EMB, and RFP was 97.7%, followed by MFX at 95.3%, LFX and para-aminosalicylic acid at 93%, and SM at 90.3%. Additionally, rifabutin and ethionamide had lower concordance rates of 67.2% and 79.1%, respectively. WGS also detected 27.9% of the PZA-related resistant mutant strains [25]. A study conducted in Thailand indicated that, except for EMB and ethionamide, WGS had good predictive diagnostic performance for resistance to INH, RFP, SM, KM, CM, para-aminosalicylic acid, ofloxacin, and LFX [26]. A study conducted in Indonesia indicated that the concordance rates of WGS and liquid cultures for RFP, INH, and SM were 93.33%, 83.33%, and 76.67%, respectively; however, it was only 63.33% for EMB. The consistency rates of second-line drugs, including AK, KM, and fluoroquinolones, were approximately 73.33–76.67% [27]. A study in Europe revealed that the predictive rates of WGS genotypic DST were as follows: RFP (97.1%), INH (96.6%), EMB (100%), LFX (83.3%), MFX (83.3%), AK (100%), KM (100%), CM (100%), prothionamide (100%), d-cycloserine (11.1%), clofazimine (20%), bedaquiline (0.0%), and delamanid (44.4%) [28].

These results demonstrate that WGS technology is effective in predicting resistance to first-line anti-TB drugs. However, the predictive effect of resistance to second-line anti-TB drugs differs among studies. The difference might be either related to the genetic differences of the *MTB* strains in different regions or to the heavy dependence on the mutation sites recruited in the data analysis for drug-resistance prediction.

The studies mentioned above suggest that WGS technology is applicable for predicting resistance to some anti-TB drugs among patients with MDR-TB or high-risk populations, but its effectiveness may be limited among general TB patients. This study is based on the TB drug-resistance survey project, in which most of the recruited patients were non-drug-resistant cases. As reported in a previous study, about 10% of RFP-resistant strains and nearly 20% of INH-resistant strains are related to other mutations [29], which are more prominent in SM and EMB. This study also indicates that WGS technology based on WHO-recommended sites can detect 60% of SM-resistant strains and 40% of EMB-resistant strains, which suggests that the gene mutations leading to drug resistance occur outside the range of the WHO-recommended sites.

For second-line anti-TB drugs, predictions based on WHO-recommended mutation sites can achieve good specificity, indicating that WGS has a good predictive effect for non-resistant strains; however, the predictive effect for resistant strains is poor. For example, our study found that in MFX-resistant strains, 53.6% of *gyrA* mutations occurred at both WHO-recommended mutation sites and other sites, whereas the remaining 46.4% of *gyrA* mutations and 7.1% of *gyrB* mutations occurred at sites outside WHO recommendations. Therefore, we conclude that if WHO-recommended mutation sites are used for drug resistance prediction, WGS can only detect drug-resistant mutations, which account for approximately 50% of the total mutations.

Similar results were observed for other second-line anti-TB drugs such as LFX, AK, KM, and CM. For example, in LFX-resistant strains, 60% of *gyrA* mutations occurred simultaneously at both WHO-recommended and other sites, whereas the remaining 40% occurred at other sites. Similar mutation-distribution characteristics were detected in *eis* and *rrs*, which were associated with resistance to SM, KM, AK, and CM, indicating that their predictive value for resistance was relatively low.

The specific distribution characteristics of mutations (Table S2) revealed that the distribution discovered by WGS varies for different mutation features. Some mutations exist in the resistant strains but not in the sensitive strains, and these mutations may be genetic variation sites closely related to resistance. Some sites are distributed in sensitive strains but not in resistant strains, and these sites may be mutations that are either phenotypically not expressed or unrelated to drug resistance. There is also a feature where mutation sites exist in both resistant and sensitive strains, and these sites may be related to cross-resistance or unrelated to resistance. The actual roles and mechanisms of these mutations are worth exploring to elucidate their relationship with drug resistance.

Additionally, in certain instances, no mutations were detected in the resistant strains. This indicates the complexity of microbial resistance mechanisms, including MTB. According to the current understanding, resistance to anti-TB drugs occurs due to genetic mutations at specific sites, and these mutations can be detected using molecular biology methods, including WGS. However, the detection of mutations is influenced by the methodology itself, including factors such as sequencing depth, the range of loci, and the number of loci considered in the predictive model analysis. Moreover, drug resistance is influenced by various factors and mechanisms, such as epigenetics, that cannot be detected through sequencing technology.

In summary, the present study demonstrated that WGS has good potential for predicting drug resistance in TB, especially for the commonly used first-line drugs INH and RFP. However, the mutation features and transmission patterns of strains may vary with region, treatment, and prevention measures. Therefore, the selection of genetic mutations may play a vital role in predicting anti-TB drug resistance using WGS. It is necessary to establish prediction models and methods that are appropriate for different regions and strains with different genetic backgrounds. The advancement of WGS methods for drug-resistance prediction will play a positive and effective role in improving the standardization of anti-TB treatment and further reducing the occurrence and spread of drug resistance due to non-standard treatment.

This study was based on a drug resistance survey of general TB patients and thus had good representativeness for general TB patients, making the study’s conclusions more applicable to the detection of anti-TB drug resistance. However, the limitations of this study include the low resistance rate among ordinary patients with TB, which resulted in a limited sample size for the resistant group. This affected the representativeness of the number and characteristics of the resistant sites presented in the study.

The present study concludes that WGS demonstrates a strong predictive capability for resistance to two essential anti-TB drugs (INH and RFP), based on the WHO-recommended mutation sites associated with resistance. This finding highlights the crucial role of WGS in promptly identifying and detecting MDR-TB. Nevertheless, the prediction of resistance to other anti-TB drugs based on the WHO-recommended mutation sites proves suboptimal. Consequently, it may be necessary to construct drug-resistance prediction models for these drugs, incorporating several additional mutation sites. As the comprehension of potential mechanisms of resistance to these anti-TB drugs deepens, the predictive value of WGS will become increasingly acknowledged and validated.

## 4. Materials and Methods

### 4.1. Study Design

A study on anti-TB drug resistance surveillance programs covering 30 of the 89 counties in Zhejiang Province was conducted from 2014 to 2018. According to the study design, all patients with positive sputum smears in designated TB hospitals after the start of the surveillance project were informed, and those that agreed to sign an informed consent form were recruited into the study. In each county, at least 30 positive *MTB* culture cases were required to be continuously recruited before the inclusion was stopped.

### 4.2. Ethics Statement

This study was approved by the Ethics Committee of the Zhejiang Provincial Center for Disease Control and Prevention. All eligible participants who agreed to participate in the program and signed an informed consent form were required to complete a questionnaire and provide at least one sputum specimen for subsequent studies.

### 4.3. Specimen Collection

Each patient was required to provide an initial sputum specimen before starting anti-TB or other relevant clinical treatments. After the sputum specimens were collected, further treatment and culture of *MTB* using solid or liquid media were carried out in the TB laboratory of each designated TB hospital. Subsequent to the culture tests, positive cultures were collected and sent to the TB-reference laboratory of the Zhejiang Provincial CDC for further DSTs.

### 4.4. Phenotypic DST

Phenotypic DSTs for anti-TB drugs were performed in the Zhejiang Provincial CDC TB laboratory by trained staff, according to standard operating procedures. The proportion method using a solid Löwenstein–Jensen medium was used. The anti-TB drugs used included INH, RFP, SM, EMB, fluoroquinolones (LFX and MFX, AK, KM, and CM. While the phenotypic drug sensitivity tests were conducted, the positive cultures of Mycobacterium were simultaneously inoculated on solid culture media containing thiophene-2-carboxylic acid hydrazide (TCH) and P-nitrobenzoic acid (PNB). Those that could grow on solid media containing TCH and PNB were regarded as NTM; otherwise, they were classified as *MTB* or *Mycobacterium bovis*. Those identified as NTM strains were excluded from this study, and the included samples for WGS were reconfirmed by the molecular method. Strains identified as NTM in the reconfirmation were also excluded from further analysis. Details of drug concentrations and testing procedures have been reported in our previous study [30].

### 4.5. WGS Genotypic DST

#### 4.5.1. DNA Isolation and Purification

*MTB* culture products were inactivated, and genomic DNA was isolated using a bacterial DNA extraction kit (QIAGEN Inc., Dusseldorf, Germany), according to the manufacturer’s instructions. The isolated and purified DNA products were transported via a cold chain to a sequencing facility. The purified genomic DNA was quantified using a TBS-380 fluorometer (Turner BioSystems Inc., Sunnyvale, CA, USA) to ensure that the DNA met the quality requirements (OD260/280 ≥1.5 and DNA quantity ≥150 ng) for library preparation, sequencing, and detection.

#### 4.5.2. Library Construction and Genome Sequencing

At least 1 μg of genomic DNA per sample was used as the input material for DNA sample preparation. The DNA samples were treated and fragmented to a size of ~400 bp. Sequencing libraries were generated using the NEXTflex™ Rapid DNA-Seq Kit following specific steps: Connect the A and B adapters; screen and remove adapter-dimer fragments; select fragments by gel electrophoresis to retain those with one end as the A adapter and the other end as the B adapter; and produce single-stranded DNA fragments by sodium hydroxide denaturation for further bridge PCR amplification. The prepared library was sequenced using the Illumina NovaSeq 6000 PE150 system (San Diego, CA92122, USA).

#### 4.5.3. Quality Control and Sample Selection

Raw sequencing data were processed using fastp (v0.20.1) [31] to remove adapter sequences and filter out low-quality bases. High-quality sequence data were then input into Kraken (v1.1.1) [32] for species identification, and samples identified as other species or with an *MTB* proportion below 80% were rejected as contaminated samples. Finally, the sequencing data from the remaining samples were aligned to the H37Rv reference genome (NC_000962.2) using BWA (v0.7.17) [33]. A total of 1105 samples with an average sequencing depth >20X and average genome coverage >95% were selected for subsequent data analysis.

#### 4.5.4. Identification of Drug Resistance-Associated Mutations

Clean sequencing data were input into the local version of TB-Profiler (v4.4.2) [34] and aligned with the reference genome of H37Rv (NC_000962.2) to identify the genotype of resistance-associated mutations and detect the resistance profile of 14 anti-TB drugs. Mutations with a frequency of less than 90% were excluded. WGS genotypic DST results were obtained based on the presence or absence of mutations in a database of drug-resistance-associated mutations with evidence levels recommended by the WHO (including Tier 1 and Tier 2 mutations) [35].

### 4.6. Statistics Analysis

R (v4.0.5) [36] was used to calculate the drug-resistance rates of each phenotypic DST. The predictive performance of WGS genotypic DST was also compared with the phenotypic DST results, including sensitivity (for the phenotypically drug-resistant *MTB* strains, the proportion of those can be judged as genetically resistant by WGS, specificity (for the phenotypically sensitive *MTB* strains, the proportion of those can be judged as genetically sensitive by GWS), positive predictive value (for the genetically resistant *MTB* strains judged by WGS, the proportion of those are considered phenotypically resistant), and negative predictive value (for the genetically sensitive *MTB* strains detected by WGS, the proportion of those are considered phenotypically sensitive). Based on the detected mutation profiles in drug resistance genes of each sample, the overall mutation profiles were classified into four categories: A for no mutation; B for only mutations other than WHO-recommended ones; C for only mutations recommended by the WHO; and D for mutations that include both WHO-recommended ones and others. The distribution of drug-resistance-associated mutations was analyzed for each drug.

## 5. Conclusions

In light of the findings from our study, we draw the conclusion that WGS is a valuable tool for identifying genome-wide variants. Leveraging the genetic variants recommended by WHO, WGS proves to be effective in detecting resistance to RFP and INH, enabling the identification of multi-drug-resistant TB patients. However, it is evident that the genetic variants recommended for predicting resistance to other anti-TB drugs require further optimization and improvement.

## Figures and Tables

**Table 1:** Phenotypic DST results.

Drug	Resistance (*n*, %)
RFP	73 (6.60)
INH	124 (11.22)
EMB	63 (5.70)
LFX	60 (5.43)
MFX	28 (2.53)
SM	159 (14.39)
AK	5 (0.05)
KM	28 (2.53)
CM	53 (4.80)

**Table 2:** Comparison between WGS genotypic and phenotypic DST.

Drug	Phenotypically Resistant (*n*, %)		Phenotypically Sensitive (*n*, %)		Sensitivity (%)	Specificity (%)	PPV (%)	NPV (%)
	Genetically Resistant	Genetically Sensitive	Genetically Resistant	Genetically Sensitive				
RFP	67 (91.8)	6 (8.2)	10 (1.0)	1022 (99.0)	91.78	99.03	87.01	99.42
INH	102 (82.3)	22 (17.7)	16 (1.6)	965 (98.4)	82.26	98.37	86.44	97.77
EMB	26 (41.3)	37 (58.7)	21 (2.0)	1021 (98.0)	41.27	97.98	55.32	96.50
LFX	36 (60.0)	24 (40.0)	9 (0.6)	1036 (99.4)	60.00	99.14	80.00	97.74
MFX	15 (53.6)	13 (46.4)	30 (2.8)	1047 (97.2)	53.57	97.21	33.33	98.77
SM	96 (60.4)	63 (39.6)	11 (1.2)	935 (98.8)	60.38	98.84	89.72	93.69
AK	3 (60.0)	2 (40.0)	0 (0)	1100 (100.0)	60.00	100.00	100.00	99.82
KM	6 (21.4)	22 (78.6)	0 (0)	1077 (100.0)	21.43	100.00	100.00	98.00
CM	3 (5.7)	50 (94.3)	3 (0.3)	1049 (99.7)	5.66	99.71	50.00	95.50

**Table 3:** Mutation distribution characteristics of anti-tuberculosis drug-resistance-associated genes.

Phenotypic DST	Group	*n*/% ^#^	RFP	INH	INH	EMB	EMB	SM	SM	SM	MFX	MFX	LFX	LFX	AK	AK	KM	KM	CM	CM
			*rpoB*	*inhA*	*katG*	*embA*	*embB*	*gid*	*rpsL*	*rrs*	*gyrA*	*gyrB*	*gyrA*	*gyrB*	*eis*	*rrs*	*eis*	*rrs*	*rrs*	*tlyA*
Resistant	A	***n***	5	104	7	0	26	48	0	0	0	26	0	56	5	0	0	0	0	0
		%	6.9	83.9	5.6	0	41.4	30.2	0	0	0	92.9	0	93.3	100	0	0	0	0	0
	B	***n***	1	7	25	62	11	105	78	149	13	2	24	4	0	2	25	25	50	53
		%	1.3	5.6	20.2	98.4	17.5	66.0	49.1	93.7	46.4	7.1	40.0	6.7	0	40.0	89.3	89.3	94.3	100
	C	***n***	55	10	16	0	20	4	0	0	0	0	0	0	0	0	0	0	0	0
		%	75.4	8.1	12.9	0	31.6	2.5	0	0	0	0	0	0	0	0	0	0	0	0
	D	***n***	12	3	76	1	6	2	81	10	15	0	36	0	0	3	3	3	3	0
		%	16.4	2.4	61.3	1.6	9.5	1.3	50.9	6.3	53.6	0	60.0	0	0	60.0	10.7	10.7	5.7	0
	Total	***n***	73	124	124	63	63	159	159	159	28	28	60	60	5	5	28	28	53	53
		%	100	100	100	100	100	100	100	100	100	100	100	100	100	100	100	100	100	100
Sensitive	A	***n***	979	910	245	1	757	216	1	0	0	989	0	959	965	0	0	0	0	0
		%	94.9	92.8	25.0	0.1	72.6	22.8	0.1	0	0	91.8	0	91.8	87.7	0	0	0	0	0
	B	***n***	43	58	733	1041	264	725	940	945	1049	86	1038	84	135	1100	942	1077	1052	1052
		%	4.2	5.9	74.7	99.9	25.4	76.6	99.4	99.9	97.4	8.0	99.3	8.0	12.3	100	87.5	100	100	100
	C	***n***	10	13	1	0	15	1	0	0	0	2	0	2	0	0	135	0	0	0
		%	0.9	1.3	0.1	0	1.4	0.1	0	0	0	0.2	0	0.2	0	0	12.5	0	0	0
	D	***n***	0	0	2	0	6	4	5	1	28	0	7	0	0	0	0	0	0	0
		%	0	0	0.2	0	0.6	0.5	0.5	0.1	2.6	0	0.7	0	0	0	0	0	0	0
	Total	***n***	1032	981	981	1042	1042	946	946	946	1077	1077	1045	1045	1100	1100	1077	1077	1052	1052
		%	100	100	100	100	100	100	100	100	100	100	100	100	100	100	100	100	100	100

^#^ ***n*** is the number of *MTB* strains and “%” refers to the related constituent ratio in each group.

## Data Availability

The raw sequence data reported in this paper have been deposited in the Genome Sequence Archive [37] in the National Genomics Data Center [38], China National Center for Bioinformation/Beijing Institute of Genomics, Chinese Academy of Sciences (GSA: CRA011960) and are publicly accessible at https://ngdc.cncb.ac.cn/gsa (Accessed from 27 July 2023).

## Supplementary Materials

The following supporting information can be downloaded at: https://www.mdpi.com/article/10.3390/antibiotics12081257/s1, Table S1: Mutation sites recommended by WHO; Table S2: Drug-resistant associated gene mutations detected by WGS.

## References

[1] Bagcchi S. (2023). WHO’s Global Tuberculosis Report 2022. Lancet Microbe.

[2] Ategyeka P.M., Muhoozi M., Naturinda R., Kageni P., Namugenyi C., Kasolo A., Kisaka S., Kiwanuka N. (2023). Prevalence and factors associated with reported adverse-events among patients on multi-drug-resistant tuberculosis treatment in two referral hospitals in Uganda. BMC Infect. Dis..

[3] Atif M., Ahmed W., Iqbal M.N., Ahmad N., Ahmad W., Malik I., Al-Worafi Y.M. (2022). Frequency and Factors Associated with Adverse Events among Multi-Drug Resistant Tuberculosis Patients in Pakistan: A Retrospective Study. Front. Med..

[4] Alvis-Zakzuk N.J., Carrasquilla M de los Á., Gómez V.J., Robledo J., Alvis-Guzmán N.R., Hernández J.M. (2017). Diagnostic accuracy of three technologies for the diagnosis of multi-drug resistant tuberculosis. Biomédica.

[5] Merker M., Egbe N.F., Ngangue Y.R., Vuchas C., Kohl T.A., Dreyer V., Kuaban C., Noeske J., Niemann S., Sander M.S. (2021). Transmission patterns of rifampicin resistant *Mycobacterium tuberculosis* complex strains in Cameroon: A genomic epidemiological study. BMC Infect. Dis..

[6] Chitpim N., Jittikoon J., Udomsinprasert W., Mahasirimongkol S., Chaikledkaew U. (2022). Cost-utility analysis of molecular testing for tuberculosis diagnosis in suspected pulmonary tuberculosis in Thailand. ClinicoEconomics and Outcomes Research.

[7] Tram T.T.B., Ha V.T.N., Trieu L.P.T., Ashton P.M., Crawford E.D., Thu D.D.A., Le Quang N., Thwaites G.E., Walker T.M., Anscombe C. (2023). FLASH-TB: An Application of Next-Generation CRISPR to Detect Drug Resistant Tuberculosis from Direct Sputum. J. Clin. Microbiol..

[8] Syed R.R., Catanzaro D.G., Colman R.E., Cooney C.G., Linger Y., Kukhtin A.V., Holmberg R.C., Norville R., Crudu V., Ciobanu N. (2023). Clinical Evaluation of the XDR-LFC Assay for the Molecular Detection of Isoniazid, Rifampin, Fluoroquinolone, Kanamycin, Capreomycin, and Amikacin Drug Resistance in a Prospective Cohort. J. Clin. Microbiol..

[9] Malinga L., Brand J., van Rensburg C.J., Cassell G., van der Walt M. (2016). Investigation of isoniazid and ethionamide cross-resistance by whole genome sequencing and association with poor treatment outcomes of multidrug-resistant tuberculosis patients in South Africa. Int. J. Mycobacteriol..

[10] Korhonen V., Kivelä P., Haanperä M., Soini H., Vasankari T. (2022). Multidrug-resistant tuberculosis in Finland: Treatment outcome and the role of whole-genome sequencing. ERJ Open Res..

[11] Penn-Nicholson A., Georghiou S.B., Ciobanu N., Kazi M., Bhalla M., David A., Conradie F., Ruhwald M., Crudu V., Rodrigues C. (2022). Detection of isoniazid, fluoroquinolone, ethionamide, amikacin, kanamycin, and capreomycin resistance by the Xpert MTB/XDR assay: A cross-sectional multicentre diagnostic accuracy study. Lancet Infect. Dis..

[12] Yadav R.N., Bhalla M., Kumar G., Sah G.C., Dewan R.K., Singhal R. (2022). Diagnostic utility of GenoType MTBDRsl assay for the detection of moxifloxacin-resistant mycobacterium tuberculosis, as compared to phenotypic method and whole-genome sequencing. Int. J. Mycobacteriol..

[13] Wu S.H., Xiao Y.X., Hsiao H.C., Jou R. (2022). Development and Assessment of a Novel Whole-Gene-Based Targeted Next-Generation Sequencing Assay for Detecting the Susceptibility of *Mycobacterium tuberculosis* to 14 Drugs. Microbiol. Spectr..

[14] Porto D.A.F.D., Monteserin J., Campos J., Sosa E.J., Matteo M., Serral F., Yokobori N., Benevento A.F., Poklepovich T., Pardo A. (2021). Five-year microevolution of a multidrug-resistant *Mycobacterium tuberculosis* strain within a patient with inadequate compliance to treatment. BMC Infect. Dis..

[15] Olawoye I.B., Uwanibe J.N., Kunle-Ope C.N., Davies-Bolorunduro O.F., Abiodun T.A., Audu R.A., Salako B.L., Happi C.T. (2021). Whole genome sequencing of clinical samples reveals extensively drug resistant tuberculosis (XDR TB) strains from the Beijing lineage in Nigeria, West Africa. Sci. Rep..

[16] Tekin K., Albay A., Simsek H., Sig A.K., Guney M. (2017). Evaluation of the BACTEC MGIT 960 SL DST Kit and the GenoType MTBDRsl Test for Detecting Extensively Drug-resistant Tuberculosis Cases. Eurasian J. Med..

[17] Srinivasan V., Ha V.T.N., Vinh D.N., Thai P.V.K., Ha D.T.M., Lan N.H., Hai H.T., Walker T.M., A Thu D.D., Dunstan S.J. (2020). Sources of Multidrug Resistance in Patients with Previous Isoniazid-Resistant Tuberculosis Identified Using Whole Genome Sequencing: A Longitudinal Cohort Study. Clin. Infect. Dis..

[18] Bainomugisa A., Lavu E., Pandey S., Majumdar S., Banamu J., Coulter C., Marais B., Coin L., Graham S.M., du Cros P. (2022). Evolution and spread of a highly drug resistant strain of *Mycobacterium tuberculosis* in Papua New Guinea. BMC Infect. Dis..

[19] Tamilzhalagan S., Shanmugam S., Selvaraj A., Suba S., Suganthi C., Moonan P.K., Surie D., Sathyanarayanan M.K., Gomathi N.S., Jayaba L. (2021). Whole-Genome Sequencing to Identify Missed Rifampicin and Isoniazid Resistance among Tuberculosis Isolates—Chennai, India, 2013–2016. Front. Microbiol..

[20] Moreno-Molina M., Comas I., Furió V. (2019). The future of TB resistance diagnosis: The essentials on whole genome sequencing and rapid testing methods. Arch. Bronconeumol..

[21] Shea J., Halse T.A., Kohlerschmidt D., Lapierre P., Modestil H.A., Kearns C.H., Dworkin F.F., Rakeman J.L., Escuyer V., Musser K.A. (2021). Low-level rifampin resistance and rpoB mutations in *Mycobacterium tuberculosis*: An analysis of whole-genome sequencing and drug susceptibility test data in New York. J. Clin. Microbiol..

[22] Yu M.C., Hung C.S., Huang C.K., Wang C.H., Liang Y.C., Lin J.C. (2022). Differential Impact of the rpoB Mutant on Rifampin and Rifabutin Resistance Signatures of *Mycobacterium tuberculosis* Is Revealed Using a Whole-Genome Sequencing Assay. Microbiol. Spectr..

[23] Finci I., Albertini A., Merker M., Andres S., Bablishvili N., Barilar I., Cáceres T., Crudu V., Gotuzzo E., Hapeela N. (2022). Investigating resistance in clinical *Mycobacterium tuberculosis* complex isolates with genomic and phenotypic antimicrobial susceptibility testing: A multicentre observational study. Lancet Microbe.

[24] Wu X., Tan G., Sha W., Liu H., Yang J., Guo Y., Shen X., Wu Z., Shen H., Yu F. (2022). Use of Whole-Genome Sequencing to Predict *Mycobacterium tuberculosis* Complex Drug Resistance from Early Positive Liquid Cultures. Teo JWP, ed. Microbiol. Spectr..

[25] Sun W., Gui X., Wu Z., Zhang Y., Yan L. (2022). Prediction of drug resistance profile of multidrug-resistant *Mycobacterium tuberculosis* (MDR-MTB) isolates from newly diagnosed case by whole genome sequencing (WGS): A study from a high tuberculosis burden country. BMC Infect. Dis..

[26] Kamolwat P., Nonghanphithak D., Chaiprasert A., Smithtikarn S., Pungrassami P., Faksri K. (2021). Diagnostic performance of whole-genome sequencing for identifying drug-resistant TB in Thailand. Int. J. Tuberc. Lung Dis..

[27] Tania T., Sudarmono P., Kusumawati R.L., Rukmana A., Pratama W.A., Regmi S.M., Kaewprasert O., Chaiprasert A., Chongsuvivatwong V., Faksri K. (2020). Whole-genome sequencing analysis of multidrug-resistant *Mycobacterium tuberculosis* from Java, Indonesia. J. Med. Microbiol..

[28] Kardan-Yamchi J., Kazemian H., Battaglia S., Abtahi H., Foroushani A.R., Hamzelou G., Cirillo D.M., Ghodousi A., Feizabadi M.M. (2020). Whole genome sequencing results associated with minimum inhibitory concentrations of 14 anti-tuberculosis drugs among rifampicin-resistant isolates of *Mycobacterium tuberculosis* from Iran. J. Clin. Med..

[29] Gupta S., Kumar C., Shrivastava K., Chauhan V., Singh A., Arora R., Giri A., Cabibbe A.M., Sharma N.K., Spitaleri A. (2022). Whole genome sequencing of isoniazid monoresistant clinical isolates of *Mycobacterium tuberculosis* reveals novel genetic polymorphisms. Tuberculosis.

[30] Liu Z., Zhang M., Wang J., Chen S., Wu B., Zhou L., Pan A., Wang W., Wang X. (2020). Longitudinal analysis of prevalence and risk factors of rifampicin-resistant tuberculosis in Zhejiang, China. BioMed Res. Int..

[31] Chen S., Zhou Y., Chen Y., Gu J. (2018). fastp: An ultra-fast all-in-one FASTQ preprocessor. Bioinformatics.

[32] Wood D.E., Salzberg S.L. (2014). Kraken: Ultrafast metagenomic sequence classification using exact alignments. Genome Biol..

[33] Li H., Durbin R. (2009). Fast and accurate short read alignment with Burrows–Wheeler transform. Bioinformatics.

[34] Phelan J.E., O’sullivan D.M., Machado D., Ramos J., Oppong Y.E.A., Campino S., O’grady J., McNerney R., Hibberd M.L., Viveiros M. (2019). Integrating informatics tools and portable sequencing technology for rapid detection of resistance to anti-tuberculous drugs. Genome Med..

[35] Walker T.M., Miotto P., Köser C.U., Fowler P.W., Knaggs J., Iqbal Z., Hunt M., Chindelevitch L., Farhat M.R., Cirillo D.M. (2022). The 2021 WHO catalogue of *Mycobacterium tuberculosis* complex mutations associated with drug resistance: A genotypic analysis. Lancet Microbe.

[36] R Core Team R (2013). R: A Language and Environment for Statistical Computing.

[37] Chen T., Chen X., Zhang S., Zhu J., Tang B., Wang A., Dong L., Zhang Z., Yu C., Sun Y. (2021). The Genome Sequence Archive Family: Toward Explosive Data Growth and Diverse Data Types. Genom. Proteom. Bioinform..

[38] CNCB-NGDC Members and Partners (2022). Database Resources of the National Genomics Data Center, China National Center for Bioinformation in 2022. Nucleic Acids Res..

