# Supplementary material for: Whole-Genome Sequencing to Predict Mycobacterium tuberculosis Drug Resistance: A Retrospective Observational Study in Eastern China
Source: Antibiotics (Basel). 2023 Jul 31;12(8):1257. doi: 10.3390/antibiotics12081257 (PMC10451829; doi:10.3390/antibiotics12081257)
Supplement: Supplementary file 1 [file antibiotics-12-01257-s001.zip › Table S2.pdf]

Table S2. Drug-resistance associated gene mutations detected by WGS

Drug	Drug-resistance associated gene mutations	Phenotypic DST		Total
		Resistant	Sensitive	
rifampicin	None	6	1022	1028
	*rpoB*_p.Asn437Asp, *rpoB*_p.Leu430Pro	1	0	1
	*rpoB*_p.Asp435Gly	1	0	1
	*rpoB*_p.Asp435Gly, *rpoB*_p.His445Asn	1	0	1
	*rpoB*_p.Asp435Tyr	1	0	1
	*rpoB*_p.Asp435Val	1	0	1
	*rpoB*_p.His445Arg	2	0	2
	*rpoB*_p.His445Asp	4	1	5
	*rpoB*_p.His445Gln, *rpoB*_p.Leu430Pro	1	0	1
	*rpoB*_p.His445Ser	1	0	1
	*rpoB*_p.His445Tyr	4	2	6
	*rpoB*_p.Leu430Pro	0	2	2
	*rpoB*_p.Leu452Pro	5	2	7
	*rpoB*_p.Ser441Leu	1	0	1
	*rpoB*_p.Ser450Leu	42	2	44
	*rpoB*_p.Ser450Phe	1	0	1
	*rpoB*_p.Ser450Trp	1	0	1
	*rpoB*_p.Val170Phe	0	1	1
isoniazid	None	22	965	987
	*inhA*_c.-154G>A	1	4	5
	*inhA*_c.-770T>C	1	0	1
	*inhA*_c.-770T>C, *katG*_p.Ser315Thr	2	0	2
	*inhA*_c.-777C>T	8	9	17
	*inhA*_c.-777C>T, *katG*_p.Ser315Thr	1	0	1
	*katG*_p.Ser315Asn	2	0	2
	*katG*_p.Ser315Thr	85	3	88
	*katG*_p.Trp198*	1	0	1
	*katG*_p.Trp477*	1	0	1
ethambutol	None	37	1021	1058
	*embA*_c.-12C>T, *embB*_p.Gly406Ala	1	0	1
	*embB*_p.Asp354Ala	1	4	5
	*embB*_p.Gln497Arg	2	1	3
	*embB*_p.Gly406Ala	3	1	4
	*embB*_p.Gly406Ala, *embB*_p.Met306Ile	2	0	2
	*embB*_p.Gly406Asp	1	1	2
	*embB*_p.Met306Ile	4	6	10
	*embB*_p.Met306Leu	1	1	2
	*embB*_p.Met306Val	11	7	18
levofloxacin	None	24	1036	1060
	*gyrA*_p.Ala90Val	11	3	14

	*gyrA*_p.Asp94Ala	1	1	2
	*gyrA*_p.Asp94Asn	7	0	7
	*gyrA*_p.Asp94Gly	15	3	18
	*gyrA*_p.Asp94Tyr	2	0	2
	*gyrB*_p.Asp461Asn	0	1	1
	*gyrB*_p.Glu501Asp	0	1	1
moxifloxacin	None	13	1047	1060
	*gyrA*_p.Ala90Val	3	11	14
	*gyrA*_p.Asp94Ala	0	2	2
	*gyrA*_p.Asp94Asn	3	4	7
	*gyrA*_p.Asp94Gly	8	10	18
	*gyrA*_p.Asp94Tyr	1	1	2
	*gyrB*_p.Asp461Asn	0	1	1
	*gyrB*_p.Glu501Asp	0	1	1
streptomycin	None	63	935	998
	*gid*_c.102delG	1	2	3
	*gid*_c.102delG, *rrs*_n.514A>C	1	0	1
	*gid*_c.115delC	2	2	4
	*gid*_p.Arg102*	1	0	1
	*gid*_p.Glu103*	1	0	1
	*gid*_p.Trp45*	0	1	1
	*rpsL*_p.Lys43Arg	72	4	76
	*rpsL*_p.Lys88Arg	9	0	9
	*rpsL*_p.Lys88Gln	0	1	1
	*rrs*_n.514A>C	7	0	7
	*rrs*_n.517C>T	2	1	3
amikacin	None	2	1100	1102
	*rrs*_n.1401A>G	3	0	3
kanamycin	None	22	1077	1099
	*eis*_c.-10G>A	1	0	1
	*eis*_c.-37G>T	2	0	2
	*rrs*_n.1401A>G	3	0	3
capreomycin	None	50	1052	1102
	*rrs*_n.1401A>G	3	0	3
